# Supplementary material for: Development and Validation of an Analytical HPLC Method to Assess Chemical and Radiochemical Purity of [68Ga]Ga-NODAGA-Exendin-4 Produced by a Fully Automated Method
Source: Molecules. 2022 Jan 15;27(2):543. doi: 10.3390/molecules27020543 (PMC8778103; doi:10.3390/molecules27020543)
Supplement: Supplementary file 1 [file molecules-27-00543-s001.zip › molecules-1524815-supplementary.pdf]

SUPPLEMENTAL DATA

Supplemental Table S1. Precision and accuracy of the calibration curves of NODAGA-exendin-4 (a) and Ga-NODAGA-exendin-4 (b) (five replicates of six standards in the expected range of concentration values).

**Reference value of concentration of NODAGA-exendin-4 [µg/mL]**	**Calculated concentration [µg/mL]**	**Calculated average concentration [µg/mL]**	**DS**	**CV%**	**Average Bias% (deviation of positive or negative from 100%)**
5	4.992<br>5.007<br>5.003<br>4.984<br>5.016	5.00	0.013	0.25	0.01
4	4.069<br>4.033<br>4.058<br>4.054<br>4.063	4,06	0.014	0.34	1.38
3.125	3.022<br>3.067<br>3.038<br>3.048<br>3.024	3,04	0.018	0.60	-2.73
1.25	1.277<br>1.272<br>1.288<br>1.280<br>1.284	1.28	0.006	0.48	2.42
1	1.004<br>1.016<br>1.024<br>1.018<br>1.028	1.02	0.009	0.88	1.79
0.75	0.730<br>0.733<br>0.730<br>0.732<br>0.730	0.73	0.001	0.16	-2.53

(a)

**Reference value of concentration of Ga-NODAGA-exendin-4 [µg/mL]**	**Calculated concentration [µg/mL]**	**Calculated average concentration [µg/mL]**	**DS**	**CV%**	**Average Bias% (deviation of positive or negative from 100%)**
5	4.985<br>5.001<br>5.005<br>5.041<br>4.977	5.00	0.025	0.50	0.04
4	4.069<br>4.038<br>4.054<br>4.062<br>4.045	4.05	0.012	0.30	1.34
3.125	3.024<br>3.059<br>3.036<br>3.060<br>3.019	3.04	0.019	0.63	-2.73
1.25	1.282<br>1.280<br>1.287<br>1.282<br>1.286	1.28	0.003	0.21	2.68
1	1.016<br>1.020<br>1.022<br>1.013<br>1.022	1.02	0.004	0.38	1.85
0.75	0.731<br>0.725<br>0.729<br>0.728<br>0.728	0.73	0.002	0.32	-2.93

(b)

Supplemental Table S2. Intra-day accuracy and precision of the proposed UV-Radio-HPLC method for NODAGA-exendin-4 (a) and Ga-NODAGA-exendin-4 (b) (n=5).

	**Reference value of concentration of NODAGA-exendin-4 [µg/mL]**	**Average concentration [µg/mL]**	**Accuracy (Bias %)**	**Precision (CV%)**
Day 1		4.69 ± 0.002	4.28	0.04
Day 2	4.50	4.69 ± 0.003	4.29	0.07
Day 3		4.69 ± 0.003	4.27	0.06
Day 1		2.02 ± 0.003	1.01	0.13
Day 2	2,00	2.02 ± 0.003	0.91	0.17
Day 3		2.02 ± 0.005	0.79	0.26
Day 1		0.81 ± 0.003	-4.34	0.40
Day 2	0,85	0.82 ± 0.003	-3.67	0.40
Day 3		0.82 ± 0.002	-3.24	0.19

(a)

	**Reference value of concentration of Ga-NODAGA-exendin-4 [µg/mL]**	**Average concentration [µg/mL]**	**Accuracy (Bias %)**	**Precision (CV%)**
Day 1		4.70 ± 0.006	4.50	0.12
Day 2	4,50	4.71 ± 0.004	4.59	0.09
Day 3		4.71 ± 0.006	4.58	0.12
Day 1		2.02 ± 0.002	1.08	0,10
Day 2	2,00	2.02 ± 0.002	1.14	0.08
Day 3		2.01 ± 0.001	0.70	0.07
Day 1		0.81 ± 0.001	-4.34	0.09
Day 2	0,85	0.81 ± 0.001	-4.43	0.09

Day 3		0.81 ± 0.001	-4.75	0.07

(b)

Supplemental Table S3. Inter-day precision and accuracy of the proposed UV-Radio-HPLC method for NODAGA-exendin-4 (a) Ga-NODAGA-exendin-4 (b) (n=15).

	**Reference value of concentration of NODAGA-exendin-4 [µg/mL]**	**Average concentration [µg/mL]**	**Accuracy (Bias %)**	**Precision (CV%)**
Day 1		4.69 ± 0.002	4.28	0.04
Day 2	4.50	4.69 ± 0.003	4.29	0.07
Day 3		4.69 ± 0.003	4.27	0.06
Day 1		2.02 ± 0.003	1.01	0.13
Day 2	2,00	2.02 ± 0.003	0.91	0.17
Day 3		2.02 ± 0.005	0.79	0.26
Day 1		0.81 ± 0.003	-4.34	0.40
Day 2	0,85	0.82 ± 0.003	-3.67	0.40
Day 3		0.82 ± 0.002	-3.24	0.19

(a)

	**Reference value of concentration of Ga-NODAGA-exendin-4 [µg/mL]**	**Average concentration [µg/mL]**	**Accuracy (Bias %)**	**Precision (CV%)**

Day 1		4.70 ± 0.006	4.50	0.12
Day 2	4,50	4.71 ± 0.004	4.59	0.09
Day 3		4.71 ± 0.006	4.58	0.12
Day 1		2.02 ± 0.002	1.08	0,10
Day 2	2,00	2.02 ± 0.002	1.14	0.08
Day 3		2.01 ± 0.001	0.70	0.07
Day 1		0.81 ± 0.001	-4.34	0.09
Day 2	0,85	0.81 ± 0.001	-4.43	0.09
Day 3		0.81 ± 0.001	-4.75	0.07

(b)

Supplemental Table S4. Radiochemical yield (RCY%), molar activity (A_m) and RCP% of three consecutive validation batches.

	RCY[%] decay corrected	A_m [GBq/µmol]	RCP[%]
**1° batch**	45.00	33.10	97.05
**2° batch**	47.30	33.30	95.75
**3° batch**	47.72	33.60	96.15
